# The Association between Ambient Air Pollution and Daily Mortality in Beijing after the 2008 Olympics: A Time Series Study

**DOI:** 10.1371/journal.pone.0076759

**Published:** 2013-10-18

**Authors:** Yang Yang, Runkui Li, Wenjing Li, Meng Wang, Yang Cao, Zhenglai Wu, Qun Xu

**Affiliations:** 1 Department of Epidemiology and Biostatistics, Institute of Basic Medicine Sciences Chinese Academy of Medical Sciences & School of Basic Medicine Peking Union Medical College, Beijing, China; 2 College of Resources and Environment, University of Chinese Academy of Sciences, Beijing, China; 3 Unit of Biostatistics, Institute of Environmental Medicine, Karolinska Institutet, Stockholm, Sweden; University of Ottawa, Canada

## Abstract

In recent decades, ambient air pollution has been an important public health issue in Beijing, but little is known about air pollution and health effects after the 2008 Beijing Olympics. We conduct a time-series analysis to evaluate associations between daily mortality (nonaccidental, cardiovascular and respiratory mortality) and the major air pollutants (carbon monoxide, nitrogen dioxide and particulate matter less than 10 µm in aerodynamic diameter) in Beijing during the two years (2009∼2010) after the 2008 Beijing Olympics. We used generalized additive model to analyze relationship between daily mortality and air pollution. In single air pollutant model with two-day moving average concentrations of the air pollutants, increase in their interquartile range (IQR) associated with percent increase in nonaccidental mortality, 2.55 percent [95% confidence interval (CI): 1.99, 3.11] for CO, 2.54 percent (95% CI: 2.00, 3.08) for NO_2_ and 1.80 percent (95% CI: 1.21, 2.40) for PM_10_, respectively; increases in the IQR of air pollutant concentrations associated with percent increase in cardiovascular mortality, 2.88 percent (95% CI: 2.10,3.65) for CO, 2.63 percent (95% CI: 1.87, 3.39) for NO_2_ and 1.72 percent (95% CI: 0.88, 2.55) for PM_10_, respectively; and increase in IQR of air pollutant concentrations associated with respiratory mortality, 2.39 percent (95% CI: 0.68, 4.09) for CO, 1.79 percent (95% CI: 0.11, 3.47) for NO_2_ and 2.07 percent (95% CI: 0.21, 3.92) for PM_10_, respectively. We used the principal component analysis to avoid collinearity of varied air pollutants. In addition, the association stratified by sex and age was also examined. Ambient air pollution remained a significant contributor to nonaccidental and cardiopulmonary mortalities in Beijing during 2009∼2010.

## Introduction

Numerous studies have linked ambient air pollution to various adverse health outcomes. Most studies were conducted in developed countries and only a small number of studies have been conducted in developing countries of Asia [1]. Large cities in China have been experiencing substantial socio-economic developments in the past three decades and often accompanied by substantial environmental pollution. Beijing, capital city of China, has had serious ambient air pollution over decades. In addition to coal combustion, rapid increase in motor vehicles has made emissions from motor vehicles another major source of air pollution [2], which has brought significant public health issues in recent years. Although several studies have examined the health consequences of air pollution in Beijing [3]–[7], most of them were conducted before the 2008 and little is known about air pollution and health outcomes after the 2008 Beijing Olympics.

In the present study, we conducted a time-series analysis to evaluate the associations between the major air pollutants, including carbon monoxide (CO), nitrogen dioxide (NO_2_) and particulate matter <10 µm in aerodynamic diameter (PM_10_), and daily mortality (nonaccidental death, cardiovascular disease and respiratory disease) in Beijing from January 1, 2009 to December 31, 2010.

## Materials and Methods

### Study Area

Beijing consists of 6 urban districts, 8 suburban districts and 2 rural counties, with a total area of 16,807.8 km^2^ and a population of 20.2 million by the end of 2011. Our study area included all these districts. But, most of previous studies in Beijing only focused on the urban areas.

### Data

Daily mortality data from January 1, 2009 to December 31, 2010 were obtained from the Chinese Center for Disease Control and Prevention (China CDC). Causes of death were coded according to the International Classification of Diseases, 10th Revision (ICD-10). The analyses were on mortalities from non-accidental causes (ICD-10:A00–R99), cardiovascular diseases (ICD-10:I00–I99), and respiratory diseases (ICD-10: J00–J98).

**Figure 1:**
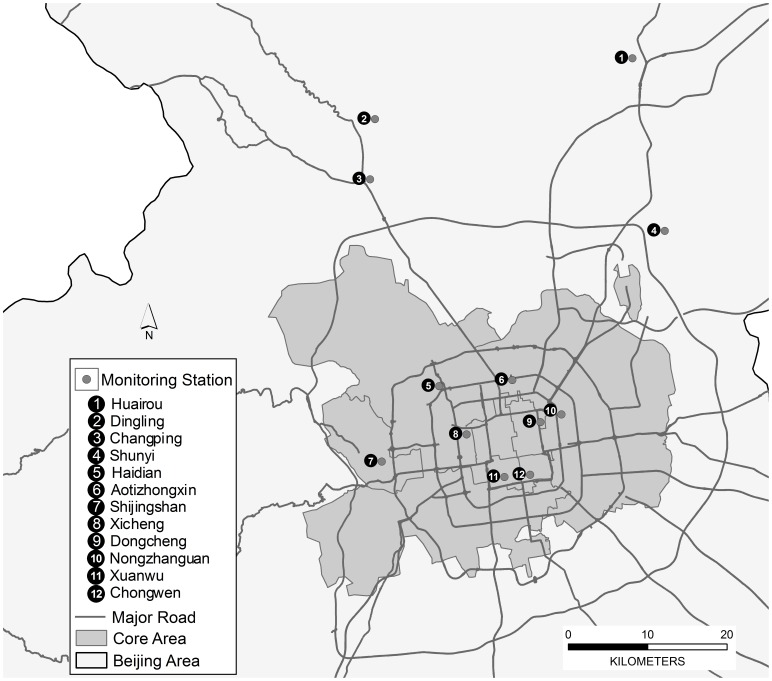
Locations of twelve monitoring stations in Beijing.

Daily air pollution data for CO, NO_2_ and PM_10_ in Beijing during the same period, were obtained from the Beijing Municipal Environmental Protection Bureau. Average daily ambient air concentrations for each pollutant were calculated based on the data from 11 of 12 available fixed-site monitoring stations in Beijing (Huairou, Dingling, Changping, Shunyi, Haidian, Aotizhongxin, Shijingshan, Xicheng, Dongcheng, Nongzhanguan, Xuanwu, Chongwen), excluding Dingling where is used as a site for background. These stations were included in the China National Quality Control for Air Monitoring Network (Figure 1), where are located away from major roads and industrial sources emissions in line with monitoring requirements.

To allow adjustment for the effect of weather conditions on mortality, we obtained data of mean daily temperature, relative humidity data and barometric pressure from the Beijing Meteorological Bureau.

### Statistical Methods

Daily number of deaths, air pollution and weather variables are linked by date and therefore can be analyzed with a time-series design [8]. We used a generalized additive model (GAM) with natural splines (NS) to analyze relationship of air pollutants, daily mortality and other covariates.

We first constructed the basic models for various mortality outcomes excluding the air pollution variables, incorporating the NS functions of time and weather conditions, which can accommodate nonlinear and monotonic relationships of mortality with time and weather variables, offering a flexible modeling tool [9]. We used different numbers of degrees of freedom (df) per year (4 df/year, 8 df/year and 12 df/year) for the time trend as sensitivity analysis. As for weather conditions, 3 df was used for temperature, relative humidity and barometric pressure [10], [11]. Day of the week was included as a dummy variable in the basic models.

Then, ambient air pollutant variables were introduced into the models and their effects on nonaccidental, cardiovascular and respiratory mortalities were examined. Briefly, we fit the following log-linear generalized additive models to obtain the estimated pollution log-relative rate *β* in Beijing:
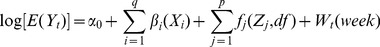
(1)


Here 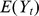 represented the expected number of deaths at day t, 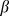 represented the log-relative rate of mortality associated with a unit increase in air pollutants, 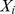 was the pollutants concentrations at day t, 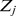 was the predictor variables other than air pollutants (i.e., time, mean daily temperature, daily relative humidity and barometric pressure ), 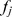 was smooth function of these variables, and 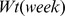 was the dummy variable for day of the week.

As single-day lag models may underestimate the cumulative effect of air pollution on mortality [12], [13], 2-day moving averages of current-day and previous-day concentrations of air pollutants (lag01) were used in our main analysis with current-day (lag 0 day) temperature, relative humidity and barometric pressure. For sensitivity analysis, we examined the effects of air pollutants with lag structures, including single-day lag (from lag 0 to lag 4) and multi-day lag [from lag0–1 day (average) to lag0–4 days (average)].

We also introduced “Principal Component” into the study and we established the model of multiple ambient air pollutants’ health effects in order to exclude the impacts of collinearity between different air pollutants in the multiple air pollutants model [14]. Information of original air pollutants were substituted by composite latent variables (principal components) in the GAM. Meanwhile, we transformed the regression coefficient *β* of the principal components into the regression coefficient *b* of the original air pollutants. Then, relative risks and their confidence intervals (CIs) were estimated to quantify the influence of each ambient air pollutant on daily mortality in multivariate model.

Finally, we conducted stratified analyses by sex (male and female) and age (45–64 years and ≥65 years) to examine potential effect modification. The 95% CI was calculated to test the statistical significance of differences between effect estimates for strata of a potential effect modifier:
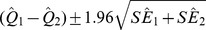
(2)


Where 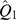 and 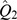 were the estimates for the two categories, and 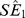 and 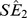 were standard errors [15].

In order to compare our results with those in other studies, the percentage increase of daily mortality associated with IQR increase in ambient air pollutant concentrations was converted into percentage increase of daily mortality associated with 10 µg/m^3^ increase for ambient air NO_2_ and PM_10_, and 1 mg/m^3^ increase for ambient air CO, respectively.

All analyses were conducted with R2.15.1 using the MGCV package [16]. The results were presented as percentage change in daily mortality per IQR increase of the three air pollutants.

## Results

A total of 152,714 deaths were recorded in Beijing between January 1, 2009 and December 31, 2010, 0.83% in those aged 0–4 years old, 5.17% in those aged 5–44 years, 19.81% in those aged 45–64 years and 74.19% in those aged 65 years or older, respectively. Overall, there were 200.4 nonaccidental deaths per day in average, including 101 from cardiovascular diseases, which accounted for 60.6% of the total of nonaccidental deaths, and 20.6 from respiratory diseases, respectively (Table 1). During our study period, the mean daily average concentrations of CO, NO_2_ and PM_10_ were 1.54 mg/m^3^, 55.02 µg/m^3^ and 121.04 µg/m^3^, respectively (Table 1). The mean daily temperature, relative humidity and barometric pressure were 13.0°C, 51% and 1012 hPa.

**Table 1:** Summary statistics of daily death numbers, air pollution concentrations and weather conditions in Beijing, China (2009∼2010).

	Mean ± SD		Min*	P(25)**	P(50)**	P(75)**	Max***
Daily deathcounts							
Nonaccidental	200.4±28.7		135	180	198	221	282
Cardiovascular	101.0±19.8		39	86	99	115	80
Respiratory	20.6±6.0		7	16	20	24	47
Air pollutants concentrations^a^							
CO (mg/m^3^)		1.54±1.01	0.27	0.87	1.24	1.84	7.79
NO_2_ (µg/m^3^)		55.02±24.04	9.90	39.27	50.41	64.36	180.67
PM_10_ (µg/m^3^)		121.04±75.30	4.91	69.00	107.00	149.00	651.18
Weather							
Temperature(°C)		13.0±11.7	−12.5	1.7	14.7	24.3	34.5
Humidity (%)		51.0±19.2	13	35	52	67	92
Barometricpressure (hPa)		1012±9.8	989.7	1004.4	1011.2	1019.2	1037.1

a Twenty-four-hour average for CO, NO_2_ and PM_10_.

* minimum.

** the 25^th^, 50^th^ (median) and 75^th^ percentile, respectively.

*** maximum.

Spearman correlation coefficients between air pollutant concentrations and weather conditions are listed in Table 2, showing close correlation between ambient air CO, NO_2_ and PM_10_ concentrations and weather conditions (all p<0.05), with the highest of ambient air CO and NO_2_ concentrations primarily from traffic sources. Ambient air pollutant concentrations significantly inversely correlated with temperature and positively with relative humidity, but weakly correlated with barometric pressure.

**Table 2:** Spearman correlation coefficients between daily air pollutant concentrations and weather conditions in Beijing (2009∼2010).

	NO_2_	PM_10_	Temperature	Humidity	Barometricpressure
CO	0.86*	0.58*	−0.33*	0.35*	0.10*
NO_2_		0.55*	−0.23*	0.27*	0.05*
PM_10_			−0.02	0.22*	−0.19*
Temperature				0.33*	−0.83*
Humidity					−0.31*

* *P*<0.05.

Estimated effects of ambient air CO, NO_2_ and PM_10_ on nonaccidental, cardiovascular and respiratory mortalities were all statistically significant in single pollutant model (Table 3). The effects decreased but remained significant after adjusting collinearity by principal component analysis (Table 3).The sensitivity analysis indicated that the increasing of the degrees of freedom led to a decrease in the effect estimates (results not shown).

**Table 3:** Percent increase of daily mortality associated with an IQR increase of CO, NO_2_ and PM_10_ with single model and principal component analysis in Beijing (mean and 95% CI), using 8 df/year.^a^

	Nonaccidental mortality	Cardiovascular mortality	Respiratory mortality
Single model			
CO	2.55 (1.99, 3.11)*	2.88 (2.10, 3.65)*	2.39 (0.68, 4.09)*
NO_2_	2.54 (2.00, 3.08)*	2.63 (1.87, 3.39)*	1.79 (0.11, 3.47)*
PM_10_	1.80 (1.21, 2.40)*	1.72 (0.88, 2.55)*	2.07 (0.21, 3.92)*
After-adjusting collinearity by principal component analysis			
CO	0.97 (0.77, 1.17)*	1.01 (0.73, 1.29)*	0.89 (0.27, 1.51)*
NO_2_	1.04 (0.82, 1.25)*	1.08 (0.78, 1.38)*	0.95 (0.29, 1.61)*
PM_10_	1.07 (0.85, 1.30)*	1.12 (0.81, 1.43)*	0.99 (0.30, 1.67)*

a We applied current-day (lag 0 day) temperature and relative humidity and 2-day moving average of air pollutant concentrations (lag01), and applied 8 df per year for time, 3 df to temperature, humidity and barometric pressure.

* *P*<0.05.

Air pollution effects on nonaccidental and cardiovascular mortality varied by sex and age (Table 4). For both males and females, effect estimates of all the three air pollutants were statistically significant. And effect estimates for females were a little bit higher than those for males, but between-sex difference was non-significant for all these three pollutants.

**Table 4:** Sex or age-specific percent increase of daily mortality of Beijing residents associated with an IQR increase of CO, NO_2_ and PM_10_, using 8 df/year.^a^

Cause of death	Proportion (%)	CO	NO_2_	PM_10_
Nonaccidental mortality				
Male	56.2	2.22 (1.47, 2.97)*	2.35 (1.62, 3.09)*	1.43 (0.63, 2.23)*
Female	43.8	2.96 (2.13, 3.79)*	2.77 (1.96, 3.59)*	2.27 (1.38, 3.17)*
45-	20.02	1.36 (0.08, 2.64)*	1.33 (0.08, 2.58)*	1.39 (0.03, 2.75)*
65-	74.42	2.70 (2.06, 3.33)*	2.71 (2.09, 3.33)*	1.82 (1.14, 2.51)*
Cardiovascular mortality				
Male	54.75	2.67 (1.63, 3.71)*	2.12 (1.10, 3.15)*	0.96 (−0.17, 2.09)
Female	45.25	3.13 (1.98, 4.28)*	3.24 (2.11, 4.37)*	2.63 (1.39, 3.86)*
45-	16.70	1.25 (−0.66, 3.16)	0.42 (−1.46, 2.30)	0.53 (−1.53, 2.59)
65-	80.77	3.11 (2.25, 3.97)*	2.97 (2.12, 3.81)*	1.97 (1.04, 2.89)*

a We applied current-day (lag 0 day) temperature and relative humidity and 2-day moving average of air pollutant concentrations (lag01), and applied 3 df to temperature, humidity and barometric pressure.

* *P*<0.05.

We observed that the effects of air pollutants were statistically significant in residents aged 65 years or older for nonaccidental and cardiovascular mortalities (Table 4), but only significant for nonaccidental mortality in residents aged 45 or older. For nonaccidental and cardiovascular mortality, effect estimates in residents aged 65 years or older for all these three air pollutants were higher than those in residents aged 45–64 years, but not reaching the level of statistically significantly difference.

**Figure 2:**
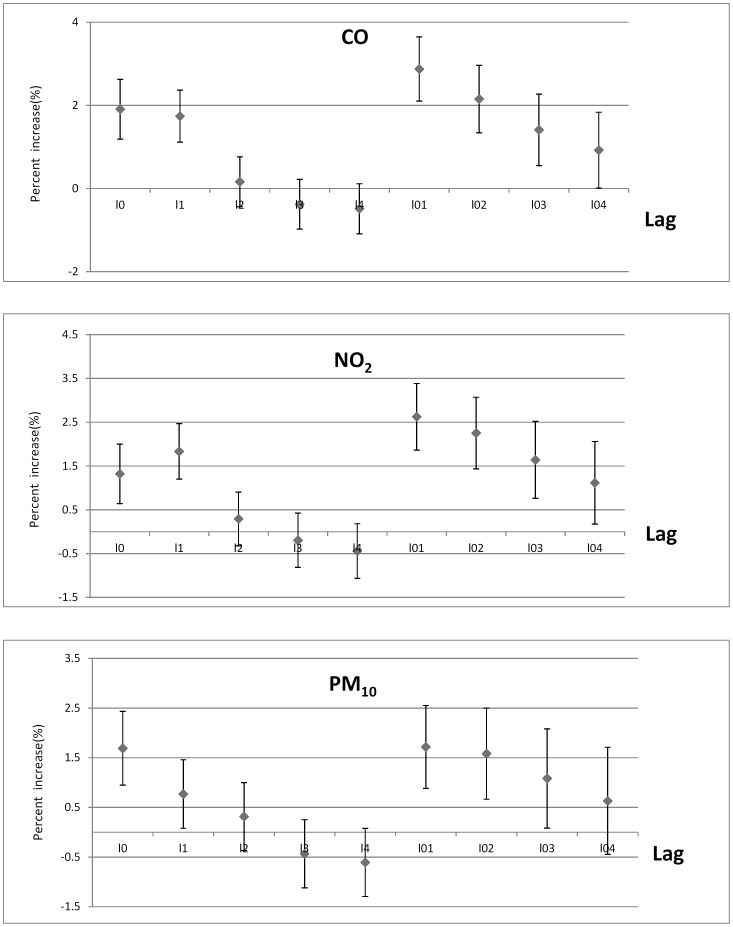
Percent increase of daily cardiovascular mortality associated with an IQR increase of CO, NO_2_ and PM_10_ concentrations, using different lag structure of pollutants.

Figure 2 presents the lag pattern of effects of air pollutants on cardiovascular mortality, with statistically significant associations for most lag days of all three pollutants except for lag3 and lag4. Generally, cumulative exposure lag01 had larger effects than single-day exposure (lag0 to lag4) and the effects of cumulative exposures (from lag01 to lag04) showed a decreasing trend.

## Discussion

The results of this time-series study found that ambient air pollutants CO, NO_2_ and PM_10_ remained strongly associated with nonaccidental deaths and mortalities caused by major cardiovascular and pulmonary diseases in Beijing after the 2008 Olympics (2009∼2010). After adjusting collinearity, all the three air pollutants were associated with nonaccidental and cardiopulmonary mortalities. Women and elderly were more vulnerable to air pollution than men and younger people.

Air pollution has been a major environmental and public health problem in Beijing for the past decades. In addition to coal combustion, the rapid increase in motor vehicles has made exhaust emissions from them another major source of air pollution [2]. In Beijing, number of automobiles increased substantially from 1.46 million in 1999 to around 5 million in 2011, which became major source of ambient air CO (86%) and NO_x_ (56%) emissions there [17], indicating Beijing still faces a substantial challenge of balancing economic growth and environment protection. Our study evaluated relationship between air pollutants and mortality after the 2008 Beijing Olympics and therefore provides important information for policy makers.

A meta-analysis of 109 time-series studies of air pollution and daily mortality, most of which were conducted in North America and Europe, indicated that 1 mg/m^3^ change of CO and 10 µg/m^3^ change of NO_2_ and PM_10_ were associated with 1.35 percent (95% CI, 0.95, 1.75), 0.61 percent (95% CI, 0.46, 0.76) and 0.64 percent (95% CI, 0.48, 0.77) increases in all daily natural deaths, respectively (in single-pollutant models) [18]. Recently, a study about the relationship between CO and daily mortality in three Chinese cities (Shanghai, Anshan, Taiyuan) estimated that 1 mg/m^3^ increase of CO was associated with 2.89 percent (95% CI: 1.68, 4.11) increase of nonaccidental mortality in the single-pollutant model [19]. Another meta-analysis of Chinese studies estimated that 10 µg/m^3^ increase in PM_10_ corresponded to a 0.44 percent (95% CI, 0.13, 0.76) increase in nonaccidental mortality [20]. There were several time series studies for association between air pollution and daily mortality in Beijing before the 2008 Olympics, Zhang et al examined the association between air pollution and daily mortality in an urban district using data from 2003 to 2008 and found that 10 µg/m^3^ increase of NO_2_ was associate with 0.271 percent (95% CI: 0.086, 0.457) increase for cardiovascular mortality and 0.947 percent (95% CI: 0.759, 1.135) increase for respiratory mortality. As for PM_10_, the corresponding figures were 0.164 percent (95% CI: 0.144, 0.184) and 0.101 percent (95% CI: 0.057, 0.145), respectively [7]. Yang et al investigated the relationship between air pollutants and cardiovascular mortality in Beijing using data from January 1, 2003 to December 31, 2003 and found that 10 µg/m^3^ increase of NO_2_ was associate with 0.404 percent (95% CI: 0.1, 0.8) increase for cardiovascular mortality and the corresponding figure was 0.381 percent (95% CI: 0.2, 0.6) for PM_10_ [21].

In order to compare our results with those in other studies, we also converting our percent increase of daily mortality associated with an IQR increase of pollutant concentrations into 10 µg/m^3^ increase for NO_2_ and PM_10_ and 1 mg/m^3^ increase for CO. Using two-day moving average of the air pollutants concentrations, an increase of 1 mg/m^3^ of CO was associated with a 3.14 percent (95% CI: 2.45, 3.83) increase in nonaccidental mortality, and the corresponding numbers were 1.25 percent (95% CI: 0.99, 1.53) for 10 µg/m^3^ increase of NO_2_ and 0.25 percent (95% CI: 0.17, 0.33) for 10 µg/m^3^ increase of PM_10_, respectively. For cardiovascular mortality, increase of 1 mg/m^3^ of CO was associated with a 3.54 percent (95% CI: 2.59, 4.49) increase, and the corresponding numbers were 1.30 percent (95% CI: 0.92, 1.68) for 10 µg/m^3^ increase of NO_2_ and 0.24 percent (95% CI: 0.12, 0.35) for 10 µg/m^3^ increase of PM_10_, respectively. For respiratory mortality, an increase of 1 mg/m^3^ of CO was associated with a 2.94 percent (95% CI: 0.84, 5.03) increase, and the corresponding numbers were 0.89 percent (95% CI: 0.05, 1.72) for 10 µg/m^3^ increase of NO_2_ and 0.29 percent (95% CI: 0.03, 0.54) for 10 µg/m^3^ increase of PM_10_, respectively_._ Our estimates on effects of gaseous pollutants (CO and NO_2_) in ambient air seemed higher than results of studies conducted before 2008 in Beijing and studies in multi-city analysis and meta analysis [7], [18], [19], [21], [22]. There are two possible explanations, automobiles became a major source of air pollutants in Beijing in the past several years. Compared with industrial emissions, automobile emission was much closer to the zones of human activities and was more likely to be inhaled. Health consequences of automobile derived from air pollutants in Beijing could be more severe and should be further investigated. Further, Beijing is also undergoing substantial changes in sociodemographic and health conditions such as rapidly aging, urbanization, lifestyles, disease spectrum, and so on, many of which may affect population susceptibility to the adverse effects of air pollution [23]. As estimated, PM_10_ concentration in Beijing is generally comparable with that in a few years ago [21] and in multi-city analysis [20] and meta-analysis [18] worldwide. Health effects of PM_10_ may be related to its composites and the residents’ sensitivity to air pollution. Components of particular mass were classified into five categories in previous studies, i.e., secondary inorganic aerosol, sea salt, heavy metal, mineral matter and construction dust [24]. For Beijing, contribution of these components to PM_2.1_ accounted for 23.9%, 6.5%, 1.1%, 13.3% and 4.0%, whereas for 12.0%, 9.5%, 0.8%, 37.1% and 7.8%, to PM_2.1-9_, respectively. The estimated contribution of anthropogenic sources to PM_2.1_ in Beijing was 3.5 times as that of nature sources whereas the same ratio was 0.6 for PM_2.1-9_. Contribution of secondary components to PM_2.1_ was equivalent to that of primary emissions, suggesting that the precursors emitted from coal combustion and vehicle exhaust should be controlled in target areas. In contrast, the ratio of primary emissions to secondary particulate matters in PM_2.1-9_ was up to 5, indicating measures are required to reduce dust from construction areas [24]. When it comes to susceptibility, the APHENA study of European and North American cities have already confirmed that the elderly and unemployed individuals were at higher risk of short-term PM exposure [25]. And obesity and active smoking have been newly recognized as a possible susceptible factors. Two cohort studies have shown that a greater body mass index enhances the susceptibility for PM-induced cardiovascular mortality, at least in women [26], [27].

In our analysis, we found associations of air pollutants and nonaccidental and cardiovascular mortalities were stronger in women than in men, which is consistent with other reports [28]. The fact that few Chinese women smoke might have further contributed to the effect of air pollutants on mortalities in China. And one study showed that effects of air pollution was greater in nonsmokers than smokers [29].

As in many other studies [30]–[32], we also found the elderly people were more vulnerable to the adverse effects of air pollution (Table 4), who have often had chronic diseases that put them be at higher risk of harmful health effects due to ambient air pollution exposure [33]. Obviously, there was an overlap between elderly people and patients with cardiopulmonary diseases. We referred to the ***Beijing Municipal Statistical Yearbook−2007*** and ***Beijing Municipal Statistical Yearbook−2010*** and found that the cardiovascular morality rates were 129.82 per 100,000 in 2007 and 156.97 per 100,000 in 2010, and the respiratory mortality rates were 58.83 per 100,000 in 2007 and 60.92 per 100,000 in 2010, respectively, indicating an increasing trend of death from chronic diseases [34], [35]. According to Beijing Statistical Bureau (2012), people over 65 years old accounted for 14.1% of the permanent residents in Beijing [36], which means that a considerable number of residents in Beijing tend to be at increased risk for air-pollution-related health effects.

Currently the most popular were single air pollutant model without considering the inner link between different air pollutants, which could lead to in certain limitations when using the single model in explanation of the results. If a multiple air pollutants were directly fitted into the model, collinearity could inevitable confound the model due to the nonindependence between different air pollutants, thus leading to the instability of the model. In order to solve the problem, principal component analysis was adopted to adjust the collinearity of different air pollutants in the multiple air pollutants model [14]. It is a multivariate method that combines three air pollutant indexes by means of an appropriate linear model, and then it generated an independent and specific composite latent variable (Principle component) with extracted variation information of original independent variable.

Sensitivity analysis of the model suggests that cumulative effects (multiday effects from lag01 to lag04) were generally larger and more sensitive than lag effects (single day effects from lag1 to lag4).

Our analysis also has several limitations. Like in most time-series studies, we used averages of monitoring stations as a surrogate for exposures to air pollution. Further, we did not have individual level information, and therefore were unable to evaluate other potential effect modifiers such as obesity and smoking. Finally, our data on weather parameters were extracted from only one monitoring station and may not accurately reflect the status of the whole city.

In summary, in our time-serious analysis, we found that ambient air pollutants were strongly associated with nonaccidental and cardiopulmonary mortalities in Beijing during 2009∼2010. Further, women and the elderly were more vulnerable to the adverse effects of ambient air pollution. These updated data in Beijing may help authorities there formulate relevant policies to improve its air environment and health status of residents.
