# Correction: The Association between Ambient Air Pollution and Daily Mortality in Beijing after the 2008 Olympics: A Time Series Study

**DOI:** 10.1371/annotation/acf66531-d1f2-4f34-b5e4-42fea77e23f1

**Published:** 2014-01-06

**Authors:** Yang Yang, Runkui Li, Wenjing Li, Meng Wang, Yang Cao, Zhenglai Wu, Qun Xu

Affiliation number 1 for the first, third, fourth, sixth and seventh authors is incorrect. The correct affiliation is: Institute of Basic Medical Sciences Chinese Academy of Medical Sciences, School of Basic Medicine Peking Union Medical College.

In addition, a funding organization was incorrectly omitted from the Funding Statement. The Funding Statement should read: "This study was funded by Gong-Yi Program of China Ministry of Environmental Protection (200909016, 201209046, 201309045) and the President Fund of UCAS (University of Chinese Academy of Sciences). URL:http://kjs.mep.gov.cn/gyxhykyzx/. The funders had no role in study design, data collection and analysis, decision to publish, or preperation of the manuscript. 

